# The Emergence of Hybrid Variants of SARS-CoV-2: Towards Hybrid Immunity

**DOI:** 10.3390/vaccines11040764

**Published:** 2023-03-30

**Authors:** Vivek P. Chavda, Suneetha Vuppu, Toshika Mishra, Pankti Balar

**Affiliations:** 1 Department of Pharmaceutics and Pharmaceutical Technology, L.M. College of Pharmacy, Ahmedabad 380009, Gujarat, India; 2 Department of Biotechnology, School of Bio Sciences and Technology, Vellore Institute of Technology, Vellore 632014, Tamil Nadu, India; 3 Pharmacy Section, L.M. College of Pharmacy, Ahmedabad 380009, Gujarat, India

In this review work, the authors emphasize the discussion on different emerging variants of SARS-CoV-2 and vaccine effectiveness against them.

Since its emergence in December 2019, Severe Acute Respiratory Syndrome Coronavirus-2 (SARS-CoV-2) is constantly evolving to project itself as a new viral variant as part of the accumulation of errors during viral replications, i.e., mutations [1,2]. The effective reproductive number of the Omicron is 3.8, which is 2.5 times higher than the Delta variant and 5–6 times higher than the other VOCs, suggesting a very high disease transmissibility rate [3]. On average, the basic reproduction number and effective reproduction number are 8.2 and 3.6, respectively [3]. In comparison to the Delta strain, Omicron has 13 times the infectivity and 2.8 times the transmissibility [4]. In the last few months, Omicron is dominating the world with high transmissibility but with less severe impacts; a recent emergence of the hybrid variant Deltacron created a buzz in the scientific community [5]. The first 158 amino acid sequences of the Delta spike and the remaining amino acids from the Omicron BA.1 spike make up the Deltacron strain. Chaitanya Kurhade and co-workers also suggested that the sera neutralization capability is 1.5-fold lower than the BA.1-spike virus [6].

The recombination of the genetic material of two different strains of SARS-CoV-2 variants in a single host as a part of co-infection leads to the emergence of hybrid (recombinant) variants of the virus [7]. This recombination may also occur in insufficiently observed populations, immunocompromised people as well as animals that serve as alternate hosts [8]. Numerous convergent evolving episodes are compatible with the high frequency of these non-synonymous mutations. Hybrid variants such as XE, XD, XG, XJ, XK, XQ and XF are currently prevalent in a society where X denotes the recombination of genetic material with two different sub-lineages of SARS-CoV-2 variants (Figure 1). Burel et al. first reported the hybrid variant of B.1.160 and Alpha variants in a lymphoma patient with severe SARS-CoV-2 infection [9]. In addition, a variation (Spike: A522S—altering viral entry and neutralization) of the B.1.1.7 lineage has emerged locally in one area. It swiftly expanded over Europe and dominated the continent. Other variations of concern (B.1.351, P.1) were discovered in low frequency in a few localities. Recombination allows for substantial, quick changes in transmissibility, severity and resistance to vaccinations and therapies, contrary to the time-consuming process of a virus alteration via errors in the reproduction of its genetic code. Studies have also identified that recombinant genomes present obstacles in computational analysis for the identification of variants. Various diagnostic techniques based on genomic analysis including nucleic acid amplification techniques, high throughput sequencing and hybrid capture-next-generation sequencing have been designed for the efficient detection of hybrid variants. Furthermore, numerous immunological and serological assays like ELISA, Chemiluminescent assays, neutralization assays, flow cytometry and electroluminescence immunoassays can be modified and improvised for rapid detection of new emerging hybrid variants [10].

Studies have reported a lower rate of replication of the virus in vaccinated individuals compared with the non-vaccinated population. The continued emergence of novel hybrids of SARS-CoV-2 is contributed to by the large number of unvaccinated populations comprising children, adults, immunocompromised individuals and aged people. The virus circulates in the non-vaccinated population which provides it conducive environment to survive, evolve and propagate [11].

Due to the worldwide expansion of SARS-CoV-2, the inability of virus- and vaccine-induced immunity to inhibit transmission, together with the formation of antigenically different variations, has prevented the achievement of herd immunity against SARS-CoV-2. According to Evans JP and co-workers [5], “Compared to the reaction to the D614G variation, neutralizing-antibody titers with mRNA vaccine against the BA.3 variant and the deltacron variant were 3.3 and 44.7 times lower, respectively.” The vaccine from Pfizer proves to be ineffective against XE variants [12]. Bates et al. demonstrated that SARS-CoV-2 infection before or after immunization offers a considerably higher increase in the neutralizing antibody response compared with two doses of vaccine alone against the different emerging viral variants [13]. To obtain the same level of humoral immunity as hybrid immunity, a third dose of vaccination is required, and hence provides a protective precedence [14]. A retrospective cohort study concluded that vaccine-induced hybrid immunity provides better protection against emerging variant-induced hospitalization than natural immunity [15]. The omicron form exhibited a significant decrease in the neutralizing capacity of both vaccination-induced and vaccine-plus-infection-induced antibodies which could indicate immunological evasion [16]. Booster dosing with mRNA vaccine demonstrated 25% more immune protection against such hybrid variants than with fully vaccinated individuals [17]. Such genetic recombination will be broadly influenced by high transmissibility and immune evasion. This action will begin in the upper respiratory tract with visible symptoms. The symptomatic and asymptomatic host can be treated with sterile immunity, comprising secretory IgA and cellular immunity, through widespread intranasal vaccination [18]. Although reports reveal that all forms of immunity diminish over time and are inefficient in protection from re-infection. However, they do lower the rates of hospitalization and the severity of the infection. The population with hybrid immunity is found to demonstrate greater protection [19].

The pandemic risk posed by the emerging new variants is the greatest obstacle in the construction of immunizations and antiviral medications with enhanced effectiveness and larger penetration. Therefore, it is crucial to develop effective strategies for tackling the concern of the new pandemic caused by emerging novel variants. Consequently, these developments will lead to advancements in the healthcare domain improving the pharmacological aspects. The improved vaccination scenario and repeated exposure to new variants will become important promoters of hybrid immunity in the global population.

## Figures and Tables

**Figure 1:**
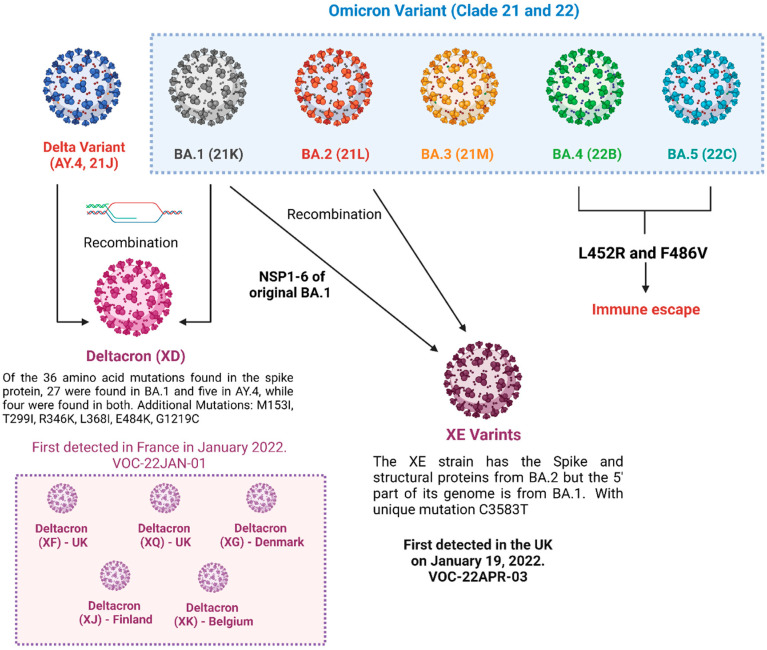
Hybrid variants of SARS-CoV-2.

## Data Availability

Not applicable.
